# Caffeine consumption disrupts hippocampal long‐term potentiation in freely behaving rats

**DOI:** 10.14814/phy2.13632

**Published:** 2018-03-07

**Authors:** J. Harry Blaise, Jee E. Park, Nicholas J. Bellas, Thomas M. Gitchell, Vy Phan

**Affiliations:** ^1^ Interdisciplinary Science Program Trinity College Hartford Connecticut; ^2^ Neuroscience Program Trinity College Hartford Connecticut; ^3^ Engineering Department Trinity College Hartford Connecticut; ^4^ Biology Department Trinity College Hartford Connecticut; ^5^ Chemistry Department Trinity College Hartford Connecticut

**Keywords:** Adenosine, Caffeine, hippocampus, LTP, plasticity, synaptic

## Abstract

Caffeine, one of the most commonly consumed psychoactive substances in the world, has long been known to alter neurological functions, such as alertness, attention, and memory. Despite caffeine's popularity, systematic investigations of its effects on synaptic plasticity in the brain are still lacking. Here we used a freely behaving rodent model of long‐term potentiation (LTP), a frequently studied form of synaptic plasticity, to assess the effects of caffeine consumption on hippocampal plasticity. LTP, which is a persistent increase in the strength of synaptic connections between neurons, is a cellular mechanism widely considered to underlie the processes of learning and memory. A group of 10‐week‐old Sprague–Dawley rats were administered caffeine (1 g/L) in their drinking water 3 weeks prior to collection of electrophysiological data. Another group of age‐matched animals received tap water and served as controls. Stimulating and recording electrodes were chronically implanted in the perforant pathway (PP) and dentate gyrus (DG) region of the hippocampus, respectively, to permit stable electrophysiological recordings of synaptic transmission at this synapse. Population spike amplitude (PSA) measures of LTP induction and duration were acquired in vivo while animals were freely behaving using a well‐established electrophysiological recording protocol. Results indicate caffeine‐treated rats (*n* = 9) had a significantly (*P* < 0.05) reduced level of LTP induction compared with controls (*n* = 10). More studies are needed to identify the exact mechanism through which caffeine alters LTP induction in this freely behaving model of synaptic plasticity.

## Introduction

Caffeine, along with theophylline and theobromine, are natural alkaloid methylxanthines that are normally found together in substances such as coffee, tea, chocolate, energy drinks, and carbonated beverages, such as cola and other soft drinks (Fredholm et al. 1999; Yoshimura 2005; Mitchell et al. 2014; McLellan et al. 2016). Caffeine behaves as a competitive antagonist of adenosine receptors, which are found throughout the brain and body and are thought to regulate the sleep‐waking cycle, the stress response, and learning and memory. The presence of caffeine inhibits intracellular enzyme phosphodiesterase, thereby preventing the conversion of cyclic AMP to noncyclic AMP. It is through this transformation that caffeine is believed to exert its influence on the sympathetic nervous system (Nehlig et al. 1992). Additionally, very high and toxic doses of caffeine increase the risk of severe ventricular arrhythmias by increasing the release of calcium from intracellular stores (Thelander et al. 2010; Wolk et al. 2012; Temple et al. 2017).

Nonetheless, caffeine remains one of the most widely consumed psychoactive substances throughout the world (Fredholm et al. 1999; Mitchell et al. 2014; McLellan et al. 2016). Average caffeine consumption is estimated at about 70–76 mg/person per day worldwide, although in many parts of the western world, including North America, Sweden, and Finland, this number rises to 210 mg/day (Fredholm et al. 1999). In today's popular culture, caffeine is widely viewed as a beneficent substance that can increase alertness and improve mood and performance (McLellan et al. 2016). Thus, an increasing number of people have become highly dependent on the drug, especially in fast‐paced societies (Heckman et al. 2010; Cappelletti et al. 2015). Indeed, many studies investigating the effects of caffeine consumption have indicated some benefits, including increased alertness, reduced symptoms of sleep deprivation and delayed onset of neurological disorders such as Alzheimer's disease, Parkinson's disease, and other age‐related declines in cognitive performance (Alhaider et al. 2010; Santos et al. 2010; Alhaider and Alkadhi 2015; Panza et al. 2015; Nehlig 2016).

When ingested, caffeine is quickly and completely absorbed into the gastrointestinal tract. The nitrogenous double‐bond ring structure of caffeine resembles that of adenosine, and studies have found that caffeine nonselectively targets four adenosine receptor subtypes, including A_1_, A_2A_, A_2B_, and A_3_ receptors (Nehlig et al. 1992; Fredholm et al. 1999; Costenla et al. 2010; Dore et al. 2011). As an adenosine receptor antagonist, caffeine tends to increase neuronal excitability which results in heightened arousal and attention (de Mendonça and Ribeiro 2001). The A_1_ receptor subtype is the primary target of caffeine and is most prevalent in the hippocampus, a region of the limbic system that has been closely linked to learning, memory, and emotion (Fredholm et al. 1999; Nehlig 1999). Indeed, removal of or damage to the hippocampus has been shown to significantly impair learning (Jarrard 1993; Bear and Malenka 1994; Eichenbaum 2001; Schiller et al. 2015).

A cellular model of learning and memory which has received a lot of attention in the scientific literature is long‐term potentiation (LTP) of synaptic transmission. High‐frequency or theta‐burst stimulation (TBS) of afferent fibers such as the perforant pathway from the entorhinal cortex has been shown to elicit robust and enduring LTP of hippocampal dentate gyrus synapses which may last for days or weeks (Bliss and Collingridge 1993; Malenka and Nicoll 1999; Blaise and Bronzino 2003; Luscher and Malenka 2012; Blaise 2013; Blaise and Hartman 2013; Blaise et al. 2015). Previous studies have examined the effects of caffeine on synaptic plasticity. In one study, caffeine injected directly into the slice chamber was found to enhance LTP in CA1 pyramidal cells synapses in vitro (Martin and Buno 2003). Brief shots of caffeine applied directly to hippocampal slices in vitro resulted in caffeine‐LTP in the Schaffer collateral‐CA1 pyramidal cell synapses. The underlying mechanisms that undergirds this form of LTP was reported to be separate from the classical NMDA‐dependent form of LTP, since previous history of LTP did not have an effect on the ability of caffeine to induce a new LTP in these same synapses.

Another study showed that caffeine enhanced basal synaptic transmission in *CA1* in vitro, but did not alter neither LTP nor paired‐pulse facilitation (a form short‐term synaptic plasticity) (Lee et al. 1987). This effect was attributed to caffeine‐induced presynaptic release of Ca^2+^ from intracellular stores which likely triggered a coincident release of neurotransmitters which are indistinguishable from those released during classical potentiation. This suggests that caffeine‐induced enhancement of basal synaptic transmission is not sufficient to induce LTP in hippocampal CA1 pyramidal cells in vitro. Another study found that caffeine facilitated LTP in CA1 in vitro (Grigoryan et al. 2012) by triggering the release of intracellular stores of Ca^2+^ which facilitates neurotransmitter release. Caffeine had a particularly potent influence on enhancing LTP in the CA2 region of the hippocampus since adenosine A1R receptors are particularly more abundant in this region (Simons et al. 2011).

There is also at least one report of chronic caffeine administration reversing stress‐induced suppression of LTP (Alzoubi et al. 2013b) by preventing decreases in phosphorylated calcium calmodulin kinase II and brain‐derived neurotrophic factors (BDNFs) in CA1 pyramidal cells. Caffeine has also been reported to preclude sleep deprivation‐induced deficits in LTP in an anesthetized model of LTP in the dentate gyrus (Alhaider and Alkadhi 2015). In that study, sleep deprivation decreased LTP which was subsequently reversed by chronic caffeine consumption putatively via caffeine‐induced enhancements in BDNF levels. Thus, caffeine appears to provide an overall neuroprotective effect to organisms exposed to chronic stressors, including sleep deprivation and chronic stress. This finding is consistent with the popular theory that drinking caffeine enhances performance on mental tasks that require attention and focus.

Despite the popularity and wide consumption of caffeine, its long‐term effects on synaptic plasticity in hippocampal circuits involved in learning and memory remain to be elucidated. Notwithstanding the previous in vitro LTP studies described above, even fewer studies have investigated caffeine's effects on a freely behaving animal model of LTP (Alhaider and Alkadhi 2015; McLellan et al. 2016). It is known that LTP studies using in vitro slice or acutely anesthetized preparations may introduce confounding factors such as lack of interconnections to other brain regions and acute anesthesia which is known to block NMDA receptor activation (a necessary component of LTP) (Duffy et al. 1981; Cain et al. 1992; Watts 2009). To avoid these issues, we systematically investigated the effects of chronic caffeine consumption on LTP in the hippocampal dentate gyrus by recording electrophysiological signals directly from the intact brains of freely behaving rats.

## Methods

All experimental protocols were performed according to the United States Public Health Service's Guide for the Care and Use of Laboratory Animals and were approved by Trinity College's Institutional Animal Care & Use Committee (IACUC). Experiments were performed on two groups (caffeine‐treated and control) of 10–17‐week‐old male Sprague–Dawley rats (300–550 g). The caffeine‐treated group received ad libitum access to 1.0 g/L caffeine in tap water for a minimum of 3 weeks prior to experimentation, whereas the control group received regular tap water (Svenningsson et al. 1999; Alhaider et al. 2010; Costenla et al. 2010). Surgical and recording procedures were performed between 8:30 and 14:00. Details of our stereotaxic surgical procedures have been published in detail elsewhere (Blaise and Bronzino 2000, 2003; Blaise et al. 2008, 2015; Koranda et al. 2011; Blaise 2013; Blaise and Hartman 2013). Briefly, rats were injected with an anesthetic cocktail containing ketamine, xylazine, and acepromazine (25 mg/kg, 2.5 mg/kg, 0.5 mg/kg, respectively). Once all muscle reflexes were lost, each rat was placed in a stereotaxic surgical frame to immobilize the head. An epoxylite‐insulated stainless‐steel bipolar stimulating electrode was inserted into the perforant pathway (AP: −8.1 mm; LAT: +4.0 mm; relative to the bregma). An epoxylite‐insulated tungsten, monopolar recording electrode was placed in the dentate gyrus (AP: −4.0 mm; LAT: +2.5 mm relative to bregma). In addition, a ground electrode and indifferent electrodes consisting of stainless‐steel machine screws were positioned equidistantly on the surface of the contralateral parietal cortex. Stimulating and recording electrodes were lowered in small increments while monitoring the evoked responses on a digital oscilloscope. The exact dorsoventral position of the electrodes was determined when the digital oscilloscope indicated a maximum triphasic waveform in the PP‐DG response. The electrodes were stabilized using dental cement and the wound was sutured using biodegradable surgical threads.

**Figure 1:**
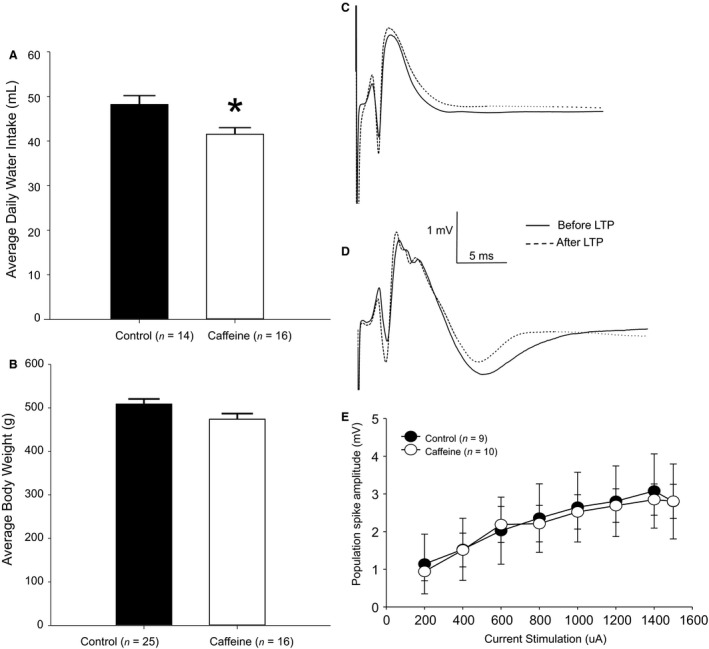
(A) Caffeine‐treated rats consumed significantly (*P* < 0.05) less water compared to controls. (B) No significant (*P* > 0.05) differences were observed in average body weight. (C) Representative evoked field potential traces in control and (D) caffeine‐treated rats. (E) No significant (*P* > 0.05) differences in the average I/O curves were observed.

After ≥5 days of postsurgical recovery, rats were acclimated to noise‐minimizing recording chambers for at least 2 h prior to recording. Then, animals were connected to low‐noise wires equipped with a commutator that allowed for free movement. Using the DAM50 differential amplifier (World Precision Instruments, Sarasota, FL) and Grass S‐88 stimulator (Astro‐Med, West Warwick, RI), biphasic square wave pulses (pulse width = 0.25 msec, 50% duty‐cycle) were used to evoke the PP‐DG response (Blaise and Bronzino 2003; Blaise et al. 2008, 2015; Blaise and Hartman 2013). The responses were amplified (gain = 1000) and bandpass‐filtered (0.1 Hz–3 KHz). The signal was recorded using a custom‐designed data acquisition analysis software system based on LabVIEW (National Instruments, Austin, TX). The software automatically extracted and quantified the population spike amplitude (PSA) measure from the evoked responses (Fig. 1). Note that the PSA measure was used exclusively as an index for quantifying LTP because PSA exhibits less variability than fEPSP measurements recorded in the dentate gyrus in freely behaving animals. Indeed, the peak fEPSP is often occluded by the superimposed population spike, such that potentiation of the population spike may be observed even in the absence of fEPSP potentiation (Taube and Schwartzkroin 1988; Bliss and Collingridge 1993).

Tetanization consisted of theta‐burst stimulation (TBS: 10 bursts of 10 pulses at 400 Hz with burst frequency of 5 Hz) at a current of intensity equal to 50% of input/output (I/O) baseline to induce LTP. After tetanization, PSA for the PP‐DG signal was calculated as the average of five responses per minute for 15 min. Subsequently, the average of 10 responses was recorded at 30, 60, 120, 180 min, and 24 and 48 h posttetanization. Posttetanization PSA values were compared to baseline for each recorded time point. The data thus obtained were subjected to a two‐way repeated‐measures analysis of variance (ANOVA) with treatment group as the between group variable and time posttetanization as the within group variable. Percent change from baseline for each time point was compared between groups. Significant main effects (*P* < 0.05) were further analyzed post hoc using the Bonferroni test to assess specific interactions between caffeine treatment and time.

## Results

In this study, we compared the effects of chronic caffeine consumption on hippocampal LTP. We observed a significant decrease (*t* = 2.7, *P* < 0.05) in average daily water intake in caffeine‐treated rats (41.5 ± 1.4 mL) compared with controls (48.2 ± 2.0 mL) (Fig. 1A). Although there was a trend toward caffeine animals (474.2 ± 12.9 g) weighing less than controls (509.1 ± 11.7 g), statistical analysis revealed no significant (*t* = 1.9, *P* > 0.05) difference in weight (Fig. 1B). Thus, although caffeine‐treated rats consumed significantly less water compared with controls, body weight remained consistent between the two groups. Perhaps, caffeine‐treated rats compensated for reduction in water intake by increased food intake. But since we did not explicitly measure food intake we cannot discount other physiological factors which may underlie these results. Even so, our findings of no significant difference in body weight are consistent with previous reports of similar lean body mass, total body fat, bone weight, and length in caffeine‐treated adult rats compared with controls (Kwak et al. 2017).

**Figure 2:**
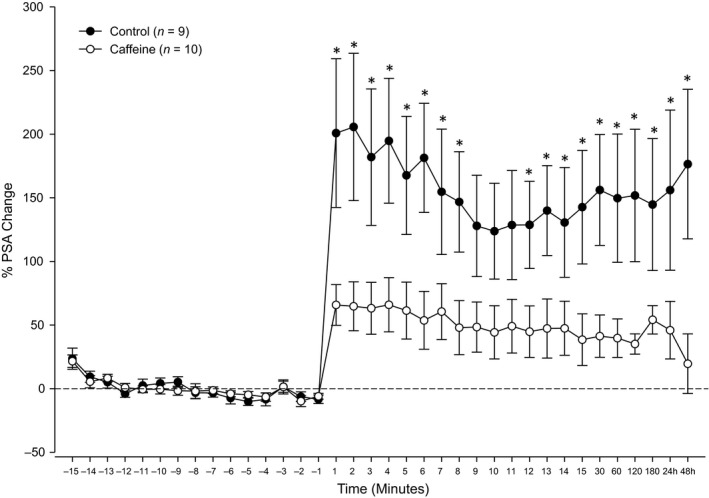
LTP was reliably and robustly induced and maintained up to at least 24 h in both caffeine‐treated and control animals. LTP was significantly lower (*P* < 0.05) in caffeine‐treated rats compared with controls at all the times tested, except at 9–11 min and 48 h posttetanization.

Evoked field potential traces recorded in control (Fig. 1C) and caffeine‐treated animals (Fig. 1D) were quantified by measuring the amplitude of the population spike and were used to establish a baseline input/output (I/O) excitability response curve for each animal prior to tetanization. As stimulation current intensity was gradually increased, both groups demonstrated a dose‐dependent increase in excitability as measured by the amplitude of the population spike (PSA) which plateaued around 1200 *μ*A (Fig. 1E). However, no significant differences (*F* = 0.21, *df* = 18, *P* > 0.05) in baseline excitability as measured by the I/O curves were observed between caffeine‐treated and control rats. After establishing a stable baseline, application of a 5‐Hz theta‐burst stimulation (TBS) protocol (10 bursts of 10 pulses delivered at 400 Hz with burst rate of 5 Hz) was used to induce LTP. As illustrated in Figure 2, LTP was reliably and robustly induced and maintained up to at least 24 h in both caffeine‐treated and control animals (*F* = 12.70, *df* = 35, *P* < 0.05). However, LTP was significantly reduced (*F* = 3.47, *df* = 35, *P* < 0.05) in caffeine‐treated rats compared with controls at all the times tested, except at 9–11 min and 48 h posttetanization. Early LTP which consists of the first 15 min immediately following TBS reached a peak of only 65.7 ± 16.0% in caffeine‐treated rats compared to 200.8 ± 58.4% in controls. While late LTP (24 h post‐TBS) persisted in both groups, it dropped to 45.9 ± 22.6% in caffeine‐treated rats compared to 155.9 ± 62.9% in controls (*P* < 0.05).

## Discussion

Three weeks prior to acquisition of electrophysiological recordings, caffeine was administered (1.0 g/L) to male rats (10–17 weeks old) through drinking water. While it had no significant (*P* > 0.05) effect on baseline excitability as indicated by the I/O curves (Fig. 1E), caffeine consumption significantly (*P* < 0.05) reduced LTP (Fig. 2). These results may reflect the upregulation of A_1_R following caffeine consumption. Indeed, this is consistent with previous reports linking caffeine to enhanced A_1_R expression which may act as a compensatory response that persists even after the cessation of caffeine treatment (Fredholm 1995; Fredholm et al. 1999). However, one previous study found a significant downregulation of A_1_R following caffeine exposure in pregnant rats and their fetuses (Leon et al. 2002). However, this discrepancy between that study and the present one may be attributable to gender differences and/or pregnancy (Turgeon et al. 2016).

Our finding of decreased LTP in caffeine‐treated rats contrasts with a previous study indicating caffeine exposure did not significantly alter LTP in the DG of anesthetized animals compared with controls (Alhaider and Alkadhi 2015). This discrepancy may likely be due to the difference in preparation (anesthetized vs. freely behaving) and/or type of stimulation (multiple high‐frequency stimulation vs. single theta‐burst stimulation). In the latter study, rats were systematically stimulated with multiple sequences of high‐frequency stimulation pulses. In contrast, this study uses the more physiologically relevant theta‐burst stimulation protocol.

NMDA receptor‐dependent LTP has two main components. First, repeated stimulation has an additive effect, driving synapses toward stable potentiation (Cain et al. 1992; Lante et al. 2006). The second component is a system in which potentiated synapses are undergirded by protein‐dependent structural changes (Luscher and Malenka 2012). A wide array of proteins and enzymatic mechanisms underlie these structural changes in the synapse, including BDNFs and antioxidant enzymes, such as superoxide dismutase (SOD) (Poo 2001; Noschang et al. 2009; Viggiano et al. 2012; Sallaberry et al. 2013; Mioranzza et al. 2014). SOD is an antioxidant enzyme that catalyzes the conversion of superoxide into hydrogen peroxide, which is then neutralized by catalase (Alzoubi et al. 2013a). Likewise, chronic caffeine consumption has been shown to increase SOD levels in the hippocampus (Noschang et al. 2009; Abreu et al. 2011). SOD plays a vital role in hippocampal LTP as previous studies have indicated its extinction alters LTP (Thiels et al. 2000; Kamsler and Segal 2003; Viggiano et al. 2012; Pomierny‐Chamiolo et al. 2013). Therefore, it is likely that our finding of reduced LTP in caffeine‐treated rats is the result of elevated levels of SOD in these animals.

On the other hand, it is important to highlight the possibility that chronic caffeine consumption may have decreased LTP by disrupting the normal sleep‐waking cycle in the caffeine‐treated rats in this study. Presumably, caffeine induces wakefulness through the blockade of A_2A_ adenosine receptors which are critically involved in the promotion of sleep (Huang et al. 2005; Lazarus et al. 2011). Unlimited access to caffeine may have disrupted the sleep‐waking cycle in these animals, which in turn may have triggered a synaptic response similar to what has been observed in animals exposed to stress. Indeed, sleep deprivation or lack of sleep, which are both considered physiological stressors, has been shown to impair LTP and, thus, may have contributed to the caffeine‐induced reduction in LTP observed in this study (Diamond and Rose 1994; Shors and Dryver 1994; Lovallo et al. 2006). In conclusion, this study examined the effects of chronic caffeine consumption on synaptic plasticity in the hippocampus in freely behaving rats. Our results indicate that caffeine has an inhibitory effect on LTP at the PP‐DG synapse which suggests that adenosine receptors may be upregulated to compensate for caffeine's blockade of adenosine. Another possibility is caffeine‐induced elevation of SOD may downregulate hippocampal LTP. A third possibility is linked to caffeine‐induced downregulation of BDNFs which may disrupt synaptic plasticity in hippocampal neuronal networks. More studies are needed to identify the exact mechanism responsible for caffeine‐induced reduction in LTP in freely behaving animals as seen in this study.

## Conflict of Interest

None declared.

## References

[phy213632-bib-0001] Abreu, R. V. , E. M. Silva‐Oliveira , M. F. Moraes , G. S. Pereira , and T. Moraes‐Santos . 2011 Chronic coffee and caffeine ingestion effects on the cognitive function and antioxidant system of rat brains. Pharmacol. Biochem. Behav. 99:659–664. 21693129 10.1016/j.pbb.2011.06.010

[phy213632-bib-0002] Alhaider, I. A. , and K. A. Alkadhi . 2015 Caffeine treatment prevents rapid eye movement sleep deprivation‐induced impairment of late‐phase long‐term potentiation in the dentate gyrus. Eur. J. Neurosci. 42:2843–2850. 26449851 10.1111/ejn.13092

[phy213632-bib-0003] Alhaider, I. A. , A. M. Aleisa , T. T. Tran , K. H. Alzoubi , and K. A. Alkadhi . 2010 Chronic caffeine treatment prevents sleep deprivation‐induced impairment of cognitive function and synaptic plasticity. Sleep 33:437–444. 20394312 10.1093/sleep/33.4.437 PMC2849782

[phy213632-bib-0004] Alzoubi, K. H. , O. F. Khabour , H. A. Salah , and B. E. Abu Rashid . 2013a The combined effect of sleep deprivation and Western diet on spatial learning and memory: role of BDNF and oxidative stress. J. Mol. Neurosci. 50: 124–133. 22956188 10.1007/s12031-012-9881-7

[phy213632-bib-0005] Alzoubi, K. H. , M. Srivareerat , A. M. Aleisa , and K. A. Alkadhi . 2013b Chronic caffeine treatment prevents stress‐induced LTP impairment: the critical role of phosphorylated CaMKII and BDNF. J. Mol. Neurosci. 49:11–20. 22706686 10.1007/s12031-012-9836-z

[phy213632-bib-0006] Bear, M. F. , and R. C. Malenka . 1994 Synaptic plasticity: LTP and LTD. Curr. Opin. Neurobiol. 4:389–399. 7919934 10.1016/0959-4388(94)90101-5

[phy213632-bib-0007] Blaise, J. H. 2013 Long‐term potentiation of perforant pathway‐dentate gyrus synapse in freely behaving mice. J. Vis. Exp. https://doi.org/10.3791/50642 10.3791/50642 PMC3992117 24327052

[phy213632-bib-0008] Blaise, J. H. , and J. D. Bronzino . 2000 Modulation of paired‐pulse responses in the dentate gyrus: effects of normal maturation and vigilance state. Ann. Biomed. Eng. 28:128–134. 10645796 10.1114/1.261

[phy213632-bib-0009] Blaise, J. H. , and J. D. Bronzino . 2003 Effects of stimulus frequency and age on bidirectional synaptic plasticity in the dentate gyrus of freely moving rats. Exp. Neurol. 182:497–506. 12895462 10.1016/s0014-4886(03)00136-5

[phy213632-bib-0010] Blaise, J. H. , and R. A. Hartman . 2013 Stimulation of perforant path fibers induces LTP concurrently in amygdala and hippocampus in awake freely behaving rats. Neural. Plast. 2013:565167. 23401801 10.1155/2013/565167 PMC3562680

[phy213632-bib-0011] Blaise, J. H. , J. L. Koranda , U. Chow , K. E. Haines , and E. C. Dorward . 2008 Neonatal isolation stress alters bidirectional long‐term synaptic plasticity in amygdalo‐hippocampal synapses in freely behaving adult rats. Brain Res. 1193:25–33. 18178177 10.1016/j.brainres.2007.11.049

[phy213632-bib-0012] Blaise, J. H. , D. N. Ruskin , J. L. Koranda , and S. A. Masino . 2015 Effects of a ketogenic diet on hippocampal plasticity in freely moving juvenile rats. Physiol. Rep. 3 https://doi.org/10.14814/phy14812.12411 10.14814/phy2.12411 PMC4463838 26009636

[phy213632-bib-0013] Bliss, T. V. P. , and G. L. Collingridge . 1993 A synaptic model of memory: long‐term potentiation in the hippocampus. Nature 361:31–39. 8421494 10.1038/361031a0

[phy213632-bib-0014] Cain, D. P. , F. Boon , and E. L. Hargreaves . 1992 Evidence for different neurochemical contributions to long‐term potentiation and to kindling and kindling‐induced potentiation: role of NMDA and urethane‐sensitive mechanisms. Exp. Neurol. 116:330–338. 1350255 10.1016/0014-4886(92)90011-e

[phy213632-bib-0015] Cappelletti, S. , D. Piacentino , G. Sani , and M. Aromatario . 2015 Caffeine: cognitive and physical performance enhancer or psychoactive drug? Curr. Neuropharmacol. 13:71–88. 26074744 10.2174/1570159X13666141210215655 PMC4462044

[phy213632-bib-0016] Costenla, A. R. , R. A. Cunha , and A. de Mendonca . 2010 Caffeine, adenosine receptors, and synaptic plasticity. J. Alzheimer's Dis. 20(Suppl 1):S25–S34. 20182030 10.3233/JAD-2010-091384

[phy213632-bib-0017] Diamond, D. M. , and G. M. Rose . 1994 Stress impairs LTP and hippocampal‐dependent memory. Ann. N. Y. Acad. Sci. 746:411–414. 7825902 10.1111/j.1749-6632.1994.tb39271.x

[phy213632-bib-0018] Dore, A. S. , N. Robertson , J. C. Errey , I. Ng , K. Hollenstein , B. Tehan , et al. 2011 Structure of the adenosine A(2A) receptor in complex with ZM241385 and the xanthines XAC and caffeine. Structure (London, England: 1993) 19: 1283–1293. 10.1016/j.str.2011.06.014 PMC3732996 21885291

[phy213632-bib-0019] Duffy, C. , T. J. Teyler , and V. E. Shashoua . 1981 Long‐term potentiation in the hippocampal slice: evidence for stimulated secretion of newly synthesized proteins. Science 212:1148–1151. 7233208 10.1126/science.7233208

[phy213632-bib-0020] Eichenbaum, H. 2001 The hippocampus and declarative memory: cognitive mechanisms and neural codes. Behav. Brain Res. 127:199–207. 11718892 10.1016/s0166-4328(01)00365-5

[phy213632-bib-0021] Fredholm, B. B. 1995 Astra award lecture. Adenosine, adenosine receptors and the actions of caffeine. Pharmacol. Toxicol. 76:93–101. 7746802 10.1111/j.1600-0773.1995.tb00111.x

[phy213632-bib-0022] Fredholm, B. B. , K. Battig , J. Holmen , A. Nehlig , and E. E. Zvartau . 1999 Actions of caffeine in the brain with special reference to factors that contribute to its widespread use. Pharmacol. Rev. 51:83–133. 10049999

[phy213632-bib-0023] Grigoryan, G. , E. Korkotian , and M. Segal . 2012 Selective facilitation of LTP in the ventral hippocampus by calcium stores. Hippocampus 22:1635–1644. 22271636 10.1002/hipo.22000

[phy213632-bib-0024] Heckman, M. A. , J. Weil , and De Gonzalez Mejia E. . 2010 Caffeine (1, 3, 7‐trimethylxanthine) in foods: a comprehensive review on consumption, functionality, safety, and regulatory matters. J. Food Sci. 75: R77–R87. 20492310 10.1111/j.1750-3841.2010.01561.x

[phy213632-bib-0025] Huang, Z. L. , W. M. Qu , N. Eguchi , J. F. Chen , M. A. Schwarzschild , B. B. Fredholm , et al. 2005 Adenosine A2A, but not A1, receptors mediate the arousal effect of caffeine. Nat. Neurosci. 8:858–859. 15965471 10.1038/nn1491

[phy213632-bib-0026] Jarrard, L. E. 1993 On the role of the hippocampus in learning and memory in the rat. Behav. Neural. Biol. 60:9–26. 8216164 10.1016/0163-1047(93)90664-4

[phy213632-bib-0027] Kamsler, A. , and M. Segal . 2003 Paradoxical actions of hydrogen peroxide on long‐term potentiation in transgenic superoxide dismutase‐1 mice. J. Neurosci. 23:10359–10367. 14614095 10.1523/JNEUROSCI.23-32-10359.2003 PMC6741007

[phy213632-bib-0028] Koranda, J. L. , D. N. Ruskin , S. A. Masino , and J. H. Blaise . 2011 A ketogenic diet reduces long‐term potentiation in the dentate gyrus of freely behaving rats. J. Neurophysiol. 106:662–666. 21613596 10.1152/jn.00001.2011 PMC3154820

[phy213632-bib-0029] Kwak, Y. , H. Choi , and J. Roh . 2017 The effects of caffeine on the long bones and testes in immature and young adult rats. Toxicol. Res. 33:157–164. 28503265 10.5487/TR.2017.33.2.157 PMC5426506

[phy213632-bib-0030] Lante, F. , de Jesus Ferreira M. C. , J. Guiramand , M. Recasens , and M. Vignes . 2006 Low‐frequency stimulation induces a new form of LTP, metabotropic glutamate (mGlu5) receptor‐ and PKA‐dependent, in the CA1 area of the rat hippocampus. Hippocampus 16: 345–360. 16302229 10.1002/hipo.20146

[phy213632-bib-0031] Lazarus, M. , H. Y. Shen , Y. Cherasse , W. M. Qu , Z. L. Huang , C. E. Bass , et al. 2011 Arousal effect of caffeine depends on adenosine A2A receptors in the shell of the nucleus accumbens. J. Neurosci. 31:10067–10075. 21734299 10.1523/JNEUROSCI.6730-10.2011 PMC3153505

[phy213632-bib-0032] Lee, W. L. , R. Anwyl , and M. Rowan . 1987 Caffeine inhibits post‐tetanic potentiation but does not alter long‐term potentiation in the rat hippocampal slice. Brain Res. 426:250–256. 3690324 10.1016/0006-8993(87)90879-1

[phy213632-bib-0033] Leon, D. , J. L. Albasanz , M. A. Ruiz , M. Fernandez , and M. Martin . 2002 Adenosine A1 receptor down‐regulation in mothers and fetal brain after caffeine and theophylline treatments to pregnant rats. J. Neurochem. 82:625–634. 12153486 10.1046/j.1471-4159.2002.01008.x

[phy213632-bib-0034] Lovallo, W. R. , N. H. Farag , A. S. Vincent , T. L. Thomas , and M. F. Wilson . 2006 Cortisol responses to mental stress, exercise, and meals following caffeine intake in men and women. Pharmacol. Biochem. Behav. 83:441–447. 16631247 10.1016/j.pbb.2006.03.005 PMC2249754

[phy213632-bib-0035] Luscher, C. , and R. C. Malenka . 2012 NMDA receptor‐dependent long‐term potentiation and long‐term depression (LTP/LTD). Cold Spring Harb. Perspect Biol. 4:https://doi.org/10.1101/cshperspect.a005710 10.1101/cshperspect.a005710 PMC3367554 22510460

[phy213632-bib-0036] Malenka, R. C. , and R. A. Nicoll . 1999 Long‐term potentiation‐a decade of progress? Science 285:1870–1874. 10489359 10.1126/science.285.5435.1870

[phy213632-bib-0037] Martin, E. D. , and W. Buno . 2003 Caffeine‐mediated presynaptic long‐term potentiation in hippocampal CA1 pyramidal neurons. J. Neurophysiol. 89:3029–3038. 12783948 10.1152/jn.00601.2002

[phy213632-bib-0038] McLellan, T. M. , J. A. Caldwell , and H. R. Lieberman . 2016 A review of caffeine's effects on cognitive, physical and occupational performance. Neurosci. Biobehav. Rev. 71:294–312. 27612937 10.1016/j.neubiorev.2016.09.001

[phy213632-bib-0039] de Mendonça, A. , and J. A. Ribeiro . 2001 Adenosine and synaptic plasticity. Drug Dev. Res. 52:283–290.

[phy213632-bib-0040] Mioranzza, S. , F. Nunes , D. M. Marques , G. T. Fioreze , A. S. Rocha , P. H. Botton , et al. 2014 Prenatal caffeine intake differently affects synaptic proteins during fetal brain development. Int. J. Dev. Neurosci. 36:45–52. 24862851 10.1016/j.ijdevneu.2014.04.006

[phy213632-bib-0041] Mitchell, D. C. , C. A. Knight , J. Hockenberry , R. Teplansky , and T. J. Hartman . Beverage caffeine intakes in the U.S. Food Chem. Toxicol. 2014;63: 136–142. 24189158 10.1016/j.fct.2013.10.042

[phy213632-bib-0042] Nehlig, A. 1999 Are we dependent upon coffee and caffeine? A review on human and animal data. Neurosci. Biobehav. Rev. 23:563–576. 10073894 10.1016/s0149-7634(98)00050-5

[phy213632-bib-0043] Nehlig, A. 2016 Effects of coffee/caffeine on brain health and disease: What should I tell my patients? Practical neurology 16:89–95. 26677204 10.1136/practneurol-2015-001162

[phy213632-bib-0044] Nehlig, A. , J. L. Daval , and G. Debry . 1992 Caffeine and the central nervous system: mechanisms of action, biochemical, metabolic and psychostimulant effects. Brain Res. Brain Res. Rev. 17:139–170. 1356551 10.1016/0165-0173(92)90012-b

[phy213632-bib-0045] Noschang, C. G. , R. Krolow , L. F. Pettenuzzo , M. C. Avila , A. Fachin , D. Arcego , et al. 2009 Interactions between chronic stress and chronic consumption of caffeine on the enzymatic antioxidant system. Neurochem. Res. 34: 1568–1574. 19283473 10.1007/s11064-009-9945-4

[phy213632-bib-0046] Panza, F. , V. Solfrizzi , M. R. Barulli , C. Bonfiglio , V. Guerra , A. Osella , et al. 2015 Coffee, tea, and caffeine consumption and prevention of late‐life cognitive decline and dementia: a systematic review. J. Nutr. Health Aging 19:313–328. 25732217 10.1007/s12603-014-0563-8 PMC12877471

[phy213632-bib-0047] Pomierny‐Chamiolo, L. , A. Moniczewski , K. Wydra , A. Suder , and M. Filip . 2013 Oxidative stress biomarkers in some rat brain structures and peripheral organs underwent cocaine. Neurotox. Res. 23:92–102. 22791409 10.1007/s12640-012-9335-6 PMC3526736

[phy213632-bib-0048] Poo, M. M. 2001 Neurotrophins as synaptic modulators. Nat. Rev. Neurosci. 2:24–32. 11253356 10.1038/35049004

[phy213632-bib-0049] Sallaberry, C. , F. Nunes , M. S. Costa , G. T. Fioreze , A. P. Ardais , P. H. Botton , et al. 2013 Chronic caffeine prevents changes in inhibitory avoidance memory and hippocampal BDNF immunocontent in middle‐aged rats. Neuropharmacology 64:153–159. 22841916 10.1016/j.neuropharm.2012.07.010

[phy213632-bib-0050] Santos, C. , J. Costa , J. Santos , A. Vaz‐Carneiro , and N. Lunet . 2010 Caffeine intake and dementia: systematic review and meta‐analysis. J. Alzheimer's Dis. 20(Suppl 1):S187–S204. 20182026 10.3233/JAD-2010-091387

[phy213632-bib-0051] Schiller, D. , H. Eichenbaum , E. A. Buffalo , L. Davachi , D. J. Foster , S. Leutgeb , et al. 2015 Memory and space: towards an understanding of the cognitive map. J. Neurosci. 35:13904–13911. 26468191 10.1523/JNEUROSCI.2618-15.2015 PMC6608181

[phy213632-bib-0052] Shors, T. J. , and E. Dryver . 1994 Effect of stress and long‐term potentiation (LTP) on subsequent LTP and the theta burst response in the dentate gyrus. Brain Res. 666:232–238. 7882033 10.1016/0006-8993(94)90777-3

[phy213632-bib-0053] Simons, S. B. , D. A. Caruana , M. Zhao , and S. M. Dudek . 2011 Caffeine‐induced synaptic potentiation in hippocampal CA2 neurons. Nat. Neurosci. 15:23–25. 22101644 10.1038/nn.2962 PMC3245784

[phy213632-bib-0054] Svenningsson, P. , G. G. Nomikos , and B. B. Fredholm . 1999 The stimulatory action and the development of tolerance to caffeine is associated with alterations in gene expression in specific brain regions. J. Neurosci. 19:4011–4022. 10234030 10.1523/JNEUROSCI.19-10-04011.1999 PMC6782739

[phy213632-bib-0055] Taube, J. S. , and P. A. Schwartzkroin . 1988 Mechanisms of long‐term potentiation: EPSP/spike dissociation, intradendritic recordings, and glutamate sensitivity. J. Neurosci. 8:1632–1644. 2896764 10.1523/JNEUROSCI.08-05-01632.1988 PMC6569208

[phy213632-bib-0056] Temple, J. L. , C. Bernard , S. E. Lipshultz , J. D. Czachor , J. A. Westphal , and M. A. Mestre . 2017 The safety of ingested caffeine: a comprehensive review. Front. Psychiatry. 8:80. 28603504 10.3389/fpsyt.2017.00080 PMC5445139

[phy213632-bib-0057] Thelander, G. , A. K. Jonsson , M. Personne , G. S. Forsberg , K. M. Lundqvist , and J. Ahlner . 2010 Caffeine fatalities–do sales restrictions prevent intentional intoxications? Clin. Toxicol. (Phila) 48: 354–358. 20170393 10.3109/15563650903586752

[phy213632-bib-0058] Thiels, E. , N. N. Urban , G. R. Gonzalez‐Burgos , B. I. Kanterewicz , G. Barrionuevo , C. T. Chu , et al. 2000 Impairment of long‐term potentiation and associative memory in mice that overexpress extracellular superoxide dismutase. J. Neurosci. 20:7631–7639. 11027223 10.1523/JNEUROSCI.20-20-07631.2000 PMC6772863

[phy213632-bib-0059] Turgeon, S. M. , S. E. Townsend , R. S. Dixon , E. T. Hickman , and S. M. Lee . 2016 Chronic caffeine produces sexually dimorphic effects on amphetamine‐induced behavior, anxiety and depressive‐like behavior in adolescent rats. Pharmacol. Biochem. Behav. 143:26–33. 26850920 10.1016/j.pbb.2016.01.012

[phy213632-bib-0060] Viggiano, A. , R. Seru , S. Damiano , B. De Luca , M. Santillo , and P. Mondola . 2012 Inhibition of long‐term potentiation by CuZn superoxide dismutase injection in rat dentate gyrus: involvement of muscarinic M1 receptor. J. Cell. Physiol. 227:3111–3115. 22015651 10.1002/jcp.23062

[phy213632-bib-0061] Watts, G . 2009 Brain connections. BMJ 338: b1317. 19366757 10.1136/bmj.b1317

[phy213632-bib-0062] Wolk, B. J. , M. Ganetsky , and K. M. Babu . 2012 Toxicity of energy drinks. Curr. Opin. Pediatr. 24:243–251. 22426157 10.1097/MOP.0b013e3283506827

[phy213632-bib-0063] Yoshimura, H. 2005 The potential of caffeine for functional modification from cortical synapses to neuron networks in the brain. Curr. Neuropharmacol. 3:309–316. 18369398 10.2174/157015905774322543 PMC2268995

